# Progression to Obesity: Variations in Patterns of Metabolic Fluxes, Fat Accumulation, and Gastrointestinal Responses

**DOI:** 10.3390/metabo13091016

**Published:** 2023-09-15

**Authors:** Fadia Milhem, Slavko Komarnytsky

**Affiliations:** 1 Plants for Human Health Institute, NC State University, 600 Laureate Way, Kannapolis, NC 28081, USA; fjmilhem@ncsu.edu; 2 Department of Food, Bioprocessing, and Nutrition Sciences, North Carolina State University, 400 Dan Allen Drive, Raleigh, NC 27695, USA; 3 Department of Nutrition, University of Petra, 317 Airport Road, Amman 11196, Jordan

**Keywords:** dietary obesity, weight gain, high-fat diet, lipid oxidation, metabolic inflexibility, metabolic rate, obesity-prone, obesity-resistant

## Abstract

Obesity is a multifactorial disorder that is remarkably heterogeneous. It presents itself in a variety of phenotypes that can be metabolically unhealthy or healthy, associate with no or multiple metabolic risk factors, gain extreme body weight (super-responders), as well as resist obesity despite the obesogenic environment (non-responders). Progression to obesity is ultimately linked to the overall net energy balance and activity of different metabolic fluxes. This is particularly evident from variations in fatty acids oxidation, metabolic fluxes through the pyruvate-phosphoenolpyruvate-oxaloacetate node, and extracellular accumulation of Krebs cycle metabolites, such as citrate. Patterns of fat accumulation with a focus on visceral and ectopic adipose tissue, microbiome composition, and the immune status of the gastrointestinal tract have emerged as the most promising targets that allow personalization of obesity and warrant further investigations into the critical issue of a wider and long-term weight control. Advances in understanding the biochemistry mechanisms underlying the heterogenous obesity phenotypes are critical to the development of targeted strategies to maintain healthy weight.

## 1. Introduction

The prevalence of obesity worldwide has increased in the last several decades indicating a global obesity trend [1]. Between 1980 and 2015, the prevalence of obesity doubled in 73 countries, largely in direct correlation with the sociodemographic development statuses of the countries studied (Global Burden of Disease study) [2]. Unexpected rapid increases in obesity prevalence were observed even in previously considered “safer” younger populations of 25-to-29-year old men residing in low-middle degree developmental countries, where the obesity rates tripled from 1.1% in 1980 to 3.8% in 2015 (NCD-RisC study) [3]. In the U.S., the National Health and Nutrition Examination Survey (NHANES) tracks similar obesity trends over a long period of time through repeated cross-sectional surveys of the population [4]. The prevalence of obesity in the U.S. was estimated to be 33.5% in men and 35.7% in women in 2005–2006 [5], and it increased to 36.5% in men and 40.8% in women in 2013–2016 [6]. In the most recently released NHANES data (2020), 42.4% of adults and 20.9% of youth are obese [6]. In some countries, obesity rates plateaued at alarming levels. One of the highest, obesity prevalence in Jordan reached 60.4% among men and 75.6% among women [7]. Another evaluation of Jordanian women age 18 and older showed that 70.6% were overweight or obese as defined by body mass index (BMI) [8]. This increase is related to modern obesogenic environments, which are influenced by the decrease in physical activity and conversions in food systems [9].

BMI, however, is a simple and convenient index to monitor the obesity that overlooks the remarkable heterogeneity of obesity when people with similar body weights or BMI can present with different sets of metabolic risk factors and comorbidities despite similar stratifications [10]. This heterogeneity presents itself as the wide spectrum response to positive energy balance [11] that incudes additional metabolically unhealthy individuals (normal weight phenotypes), obese individuals with no associated metabolic risk factors (metabolically healthy phenotypes), extreme obesity (super-responder phenotypes), and healthy individuals that resist obesity despite the obesogenic environment (obesity resistant or non-responder phenotypes) [12]. The diversity of the obesity phenotypes could be explained in part by differences in visceral (central, abdominal) adipose tissue distribution and ectopic fat accumulation that are not captured by BMI [13]. While a general measure of visceral obesity can be estimated by taking the waist circumference (a single threshold of 102 cm in men and 88 cm in women in the U.S. or 94 cm for men and 80 cm for women in Europe), more advanced analyses, such as MRI and CT scan, are often necessary [14]. Visceral obesity follows the general obesity trends; in U.S. adults it increased from 29.1% for men and 46.0% in women in 1988–1994 to 42.0% for men and 61.5% for women in 2009–2010 [15]. However, visceral obesity is an independent marker of morbidity and mortality [16] and is associated with metabolic risk factors, such as hyperlipidemia, cardiovascular diseases, and systemic inflammation [17]. These effects may be mediated by a variety of pathophysiological abnormalities, including changes in fat deposition, adipocyte function, inflammatory and adipokine responses, and insulin resistance [13]. It is presently not clear how visceral obesity and associated metabolic dysregulation contribute to the development of the vast spectrum of obesity phenotypes; however, it is generally accepted that it adds to the unexpected observations that obesity may differently affect mortality risks in chronic disease states and fit obese individuals, with overweight status being somewhat protective in normal populations, and metabolically healthy obese states being advantageous over the spectrum of other obesity-related outcomes [18]. 

The variety of individual responses to obesogenic environment and the onset of obesity and their relationship to various health outcomes are not fully understood. Apart from the compounding genetic and lifestyle variables, two parameters are considered as the main contributors to an individual’s response to obesity: metabolic perturbations in visceral fat alongside ectopic accumulation of fat in the liver and enhanced inflammatory responses associated with visceral fat activation. Our understanding of the altered cellular metabolism, often driven by mitochondrial dysfunction to produce metabolic disparity, which in turn influences inflammation and energy balance in the metabolically active tissues, is critically lacking. These areas are the focus of the present review. 

## 2. Progression to Obesity

Adipose weight gain may appear at any age with certain trends in different age groups, including children, adolescents, and adults, with differences between genders [19]. It is modulated through dietary intake, physical activity, and metabolism, all which are affected by genetic traits [20]. Socioeconomic influences and social vulnerability further affect obesity rates in communities [21]. Adolescence obesity commonly occurs before the age of 5 and continues into adulthood, specifically if one of the parents is obese [22]. The risk of developing obesity in early life can also be influenced by the mother’s excessive weight, high BMI, and type 2 diabetes mellitus during pregnancy [23]. The weight trajectory varies between women and men, with women experiencing more fluctuations throughout their lives [24]. There are at least three developmental stages at which women are at greater risk to gain adipose tissue, after puberty starts [25], after their first pregnancy [26], and when they reach menopause [27]. Research studying male obesity is not widespread due to a variety of social and cultural stigmas [28]. 

Progression to obesity is ultimately linked to the overall energy balance of the body when energy intake (EI) exceeds energy expenditure (EE) [29]. The excess energy is deposited in body tissues, including conventional adipose depots as well as ectopic accumulation in the non-target organs, such as liver and muscle [30], based on the individual hypothetical body weight set points and patterns of fat accumulation. 

### 2.1. Calorie Intake

An important factor in the control of body weight is dietary energy density (DED) as it correlates with increase in body weight [31]. Energy density levels range from 0 kcal/g to 9 kcal/g and are determined by the macronutrient composition and moisture content of meals and drinks, with pure carbohydrate or protein (4 kcal/g each), ethanol (7 kcal/g), and fat (9 kcal/g) being the major sources of caloric intake [32]. However, energy intake does not essentially represent total energy absorption [33]. For this reason, the previously reported Wishnofsky guideline that recommends achieving a daily 500–750 kcal deficit to support adult weight loss [34] may no longer hold true as it does not account for either catabolism-induced differences in energy expenditure among macronutrients [35] or the modern shift towards energy-dense foods depleted of essential nutrients known to act against metabolic disorders [36]. At the same time, a constant error of 175 kcal energy intake per day may translate to 3 kg body weight gain or loss per year, suggesting that such a small margin of error cannot be fully controlled at the individual level [37].

Even though twin studies suggested significant heritability for the amounts of foods and fluids ingested, short-term energy intake appears to be unregulated and varies spontaneously within a relatively wide range [38]. However, long-term trends in body weight changes lead to alterations in physiology and the nature of the food consumed to replace loss or avoid gain, and this phenomenon shows high individual variability in the range of 40% to 100% energy compensation [39]. This compensation is not immediate as it is not observed after 1–2 days following the deviation from the average energy intake but reaches significance after 3–4 days of observations [40]. 

### 2.2. Energy Expenditure

The second major variable in the relationship of obesity with energy imbalances is energy expenditure [41]. Daily energy expenditure varies based on basic metabolic rate, type, and duration of physical activity, sleep patterns, and variations in body temperature. The compounding nature of energy expenditure mechanisms includes resting metabolic rate (RMR, cellular metabolism, and digestion processes), non-exercise activity (NEAT, including habitual sitting, standing, walking, and social interaction activities), low-intensity and high-intensity work or exercise-related activity, and environment-related adaptive thermogenesis [42]. While generally believed that changing levels of physical activity could equilibrate the energy intake and promote a negative energy balance, the scale of the compensatory mechanisms is often overlooked. For example, an adult consuming 290 kcal meal is expected to walk for around 90 min or 5 km to regain the hypothetical net energy balance [43]. For this reason, the direct relationship between obesity and physical activity in long-term longitudinal studies is not as prominent [44,45]. Significant associations between physical activity with incident obesity were reported in three subsequent longitudinal studies published after 2012 [46]. 

Energy expenditure was found in the reciprocal compensatory adaptation loop with dietary intake [47]. First, habitual NEAT may decrease in response to increased exercise, especially if it is associated with fatigue or discomfort [48]. Second, exercise may be associated with at least a partial compensatory increase in energy intake [49], although high-intensity exercise may have the opposite effect due to reduced appetite and delayed gastric emptying [50]. These effects may also correlate to individual differences in carbohydrate and fat metabolism as at least one study showed lower energy intake in subjects with higher fat oxidation rates [51]. Third, reduced energy intake in the form of caloric restriction is generally associated with lower physical activity [52]. However, the overall mismatch in net energy balance does not continue indefinitely and at some point, increased energy expenditure always results in an increase in energy intake as a protective adaptation for long-term body weight preservation [53]. 

### 2.3. Obesity as Body Weight Set Point

Typical variance in individual body weights is very small over both short-term (0.5% over 6–10 weeks) and long-term, even when associated with chronic metabolic disorders (3–4% over 5 years) [54]. The observed weight stability is suggestive of homeostatic control, colloquially defined as a body weight “set point” (or settling point). Resting metabolic rate consisting of energy expended from skeletal muscles and the digestive tract comprises near 70% of total energy expenditure and has a major impact on the dynamic equilibrium of weight maintenance [55]. Thermic effect of physical activity of the skeletal muscles (10–20%) and the adaptive thermogenesis in skeletal muscles and brown adipose tissue (5–10%) form secondary streams of the total energy expenditure. In the resting state, 70% of energy is generated via mitochondrial ATP production, 20% as compensation for proton leakage and 10% is non-mitochondrial [56]. However, obese states have higher basal metabolism than lean states, suggesting that adipose tissue also contributes to energy expenditure [57]. When food intake was restricted, a reduction in metabolic rate and heat production occur to facilitate a return to the set weight [58]. The set weight also seems to increase annually at the small average rate of 0.2–0.3 kg per year, and considering the large amount of calories consumed at the same time, other physiological factors must be considered at least partially responsible for this increase [39]. Additionally, nearly 20% of subjects showed little body weight change over the observed period, suggesting high individual differences and the existence of non-responders that manage to maintain their body weight [59]. 

Such a nearly precise control of body weight over long periods of time then questions the notion that the onset and progression to obesity are major disturbances of metabolic homeostasis. The emerging evidence suggests that the primary inability to maintain weight is linked to the rate of replacement of energy lost due to caloric restriction (dieting) or increased physical activity (expenditure). In obese states, the reduction of caloric intake was not compensated only when low-energy dense foods were consumed [60]. In other words, high-energy dense foods delayed satiety and allowed for overcompensation, even if similar amounts of foods were consumed [61]. 

As such, a variety of fasting forms and food restriction practices will be effective for promoting initial weight loss [62] but will fail to prevent the long-term compensatory mechanisms from defending the overweight set point and returning to it within 1–3 years [63]. Modulation of individual socioeconomic choices to consume certain types of food and at a certain frequency are often routed in the activity of several neurophysiological factors that arise from dopamine, opioid, endocannabinoid, and melanocortin regulatory networks [64], which add an additional level of complexity in the attempt to understand individual variability under ad libitum food intake. 

### 2.4. Utilization of Nutrients

Human subjects spending several days in the metabolic chambers typically show low variations in energy expenditure (2%) and high variations in food intake (20%) [65], thus pointing at dietary intake as a more critical individual determinant of energy homeostasis. From these numbers, it is also reasonable to conclude that (i) variations in the resting metabolic rate alone are not sufficient to justify the onset and progression to obesity, and (ii) failure to adjust food intake to the current energy expenditure must be the major culprit.

Three macronutrients found in all foods in different proportions have different efficiencies of nutrient utilization, with different fats (2–3%), carbohydrates (6–8%), and proteins (25–30%) associated with energy losses due to differences in their metabolism and storage [66]. Digestion of a carbohydrate meal and extra glucose appearance in the bloodstream stimulates glucose uptake and oxidation in the insulin-sensitive tissues coupled with a simultaneous suppression of lipolysis and lipid oxidation [67]. All dietary glucose is typically oxidized within 24 h of intake (glycolysis to acetyl-CoA that enters Krebs cycle) due to the limited capacity of body carbohydrate storage in the form of glycogen [68] and a very limited ability of human tissues to metabolize glucose into fatty acids via de novo lipogenesis (2–4%) [69]. Thus, dietary carbohydrate will favor suppression of fat metabolism and fat deposition into the adipose tissue via insulin signaling [70]. Early insulin response (30 min after food ingestion) rather than maximum insulin response seems to better predict changes in fat mass and body weight gain [71]. 

The macronutrient capacity to promote satiety follows a similar pattern (protein > carbohydrate > fat), with fats typically more likely to promote overconsumption of energy and the subsequent weight gain. However, subjects with increased capacity to oxidize fat appear to gain little weight under these conditions and therefore, present with a non-responder phenotype [72]. A hidden variable in this progressive transformation towards increased fat production and storage is the fact that it requires higher energy demands to support the additional adipose tissue as well as larger lean body mass to sustain a similar level of physical activity. Together, these compensatory body weight gains may result in a new setpoint that is defended as a part of the revised homeostatic control. 

One of the key substrates that connects utilization of macronutrients is oxaloacetate, a Krebs cycle intermediate. Oxaloacetate is typically regenerated within the Krebs cycle; however, this molecule forms a core metabolism node that connects to gluconeogenesis and the regeneration of glucose in the liver from non-carbohydrate substrates, such as amino acids and glycerol, during prolonged fasting or diabetes [73]. When dietary intake is balanced, oxaloacetate is replenished in the liver through glycolysis glucose > pyruvate > oxaloacetate to support normal fatty acid oxidation within the Krebs cycle. In the adipose tissue, oxaloacetate can also exit mitochondria via interconversion to citrate, and the cytosolic oxaloacetate is then used for fatty acid synthesis. Oxaloacetate also participates in the reduction of elevated levels of free fatty acids by conversion to phosphoenolpyruvate and glycerol that is used for their re-esterification to triglycerides [74]. Therefore, when oxaloacetate is excessively used in other metabolic pathways, its concentration in the Krebs cycle decreases to the point that acetyl-CoA can be no longer used for energy production. In this instance, acetyl-CoA is diverted to the ketogenic formation of acetoacetate, hydroxybutyrate, and acetone [75]. A similar metabolic shift is also observed during the intermittent fasting [76], although an 18 h fast is often not sufficient to fully deplete the glycogen stores [77]. Finally, depleted oxaloacetate levels can be replenished via consumption of amino acids derived from proteins and catabolism of lean body mass. 

Several Krebs cycle metabolites can exit mitochondria, including citrate and malate. Plasma citrate levels are typically elevated in obesity, and inhibition of the mitochondrial citrate carrier, Slc25a1, reverts steatosis, glucose intolerance, and inflammation in metabolic disorders [78]. Citrate also plays an important role in the reprogramming of metabolic pathways upon activation of immune cells and onset of inflammation [79], although exact molecular mechanisms and effector functions of Krebs cycle metabolites in the pathophysiology of obesity remain unknown. 

### 2.5. Macronutrient Selection and Weight Control

To better understand how macronutrients interact to maintain a stable body weight, we need to evaluate the typical energy stores available to the body and how newly ingested energy is partitioned into these stores (Table 1). In healthy states, only 5 g of free glucose are present in the bloodstream and 15–25 g in the whole body, which are negligible amounts when compared to 200–350 g of daily carbohydrate intake. Since glucose is oxidized at the maximum rate of 10 g/h, most of the ingested carbohydrate must be imported into the metabolically active tissues and stored as glycogen [80]. The body glycogen reserve is therefore similar to a daily carbohydrate intake and is tightly regulated; thus, carbohydrates are immediately and preferentially metabolized when available. 

In contrast, dietary fat is primarily targeted for deposition. This is achieved via a series of hydrolysis and re-esterification steps during which triglycerides are broken down to free fatty acids and glucose in the intestine, reassembled in the intestinal cells, secreted into the mesenteric lymphatic system, hydrolyzed on the surface of the epithelial cells of the adipose tissue capillaries, and re-esterified once again when they enter adipocytes for storage [81]. Insulin secreted in response to the carbohydrate load of the mixed meal further facilitates fat storage and decreases fat oxidation. Since the insulin-signaling network is primarily responsible for the precise regulation of carbohydrate metabolism, its indirect effects on lipid metabolism are not expected to be exact or follow predictable compensation patterns. For this reason, errors of fat metabolism are believed to be the primary determinants of long-term energy balance and should be thoroughly investigated for their relationships to individual variability and progression to obesity. 

When compared with fat energy reserves typically available to the body (Table 1), short-term effects of high-fat meals are expected to be negligible. Long term, however, the body responds to excessive fat intake by expanding its capacity to store fat by increasing the number of adipocytes in the various adipose tissue depots. This gradual expansion is expected to continue until a sufficient amount of adipose tissue is developed to successfully match the current fat intake to the appropriate fat oxidation rates that ensure a new equilibrium. The long-term nature of this process holds the potential to amplify even minor individual differences in lipid metabolism and adipocyte differentiation to achieve substantial variability and the emergence of both super-responder and non-responder phenotypes. Substantial physical activity may counteract these effects by increasing substrate oxidation in muscle and post-activity depletion of glycogen reserves both in muscle and liver, with the liver shift from a glucose-removing to a glucose-producing state being an important checkpoint for changes in the overall energy balance. 

## 3. Pathophysiology of Body Fat Distribution

Adipose weight gain may appear at any age with certain trends in different age groups, including major developmental shifts, such as puberty, adolescence, pregnancy, menopause, and normal physiological aging. Adipose tissue tends to increase in middle age and decrease in the elderly, and this change is often accompanied by fat redistribution from subcutaneous tissue to abdominal and ectopic locations [82]. The wide range of individual obesity phenotypes presented by these populations as well as the increased risk of developing insulin resistance that is associated with both excessive and insufficient fat mass [83] suggests that intrinsic differences between adipose tissue depots may be in part responsible for this variation. 

### 3.1. Patterns of Fat Accumulation

Unequal distribution of the adipose tissue among individuals paired together with different levels of ectopic fat deposition and inflammatory dysregulation add another level of complexity to homeostatic control of the body weight [13]. Different locations and/or rates of the adipose tissue expansion suggests the maximum capacity of adipose tissue expansion as another individualize trait, possibly under genetic control [84]. Reginal fat distribution is strongly influenced by genetic factors as shown in young and elderly twins [85]. Additionally, in GWAS studies, several developmental genes have been suggested to correlate with adiposity and fat distribution, including white fat adipogenesis and browning of the adipose tissue [86]. 

The major striking difference between individual fat depots is observed in the subcutaneous versus visceral (intraabdominal) adipose tissue, where transplantation of the subcutaneous fat into the visceral cavity improves body weight, adiposity, and carbohydrate metabolism but not vice versa [87]. Increased visceral fat is also associated with a greater risk of type 2 diabetes in obese but otherwise healthy subjects [88]. Individual variations in visceral fat accumulation may depend on gender and ethnicity [89]. The molecular mechanisms responsible for these genetic effects are largely not determined at the present time. Overall, around 80–90% of adipose tissue is accumulated in subcutaneous depots, including the abdominal, subscapular, gluteal, and femoral areas [90]. Females have significantly greater mean anterior and posterior subcutaneous fat than in males. However, males possess more posterior fat than anterior fat in their body, whereas women have no difference between posterior and anterior fat distribution [91]. Comparing individuals of similar weight and age, females have an additional 22 mm of subcutaneous fat mainly accumulated in the breasts and posterolateral sites [92]. Although women have higher subcutaneous fat accumulation and men accumulate more visceral fat, estrogen decrease post menopause causes an increase in visceral fat [93]. On the contrary, lower body subcutaneous fat is associated with a lower risk of metabolic and cardiovascular complications, possibly due to increased capacity to support the triglyceride clearance, a healthier lipid profile, and a healthier energy reserve that protects the body from lipotoxicity [94]. 

Both dietary change and aerobic exercise (walking, running, swimming) were correlated with weight loss and preferential loss of visceral fat; however, the correlation for diet was strong (R^2^ = 0.737, *p* < 0.001) and the one for exercise was modest (R^2^ = 0.451, *p* < 0.001) [95]. This effect may be partially responsible for the success of surgical therapies, such as gastric bypass or gastric sleeve to preferentially reduce visceral fat by 40% as they typically lead to substantial dietary changes [96]. Prolonged sedentary patterns associated with watching TV/video are more likely to promote visceral adiposity and increase metabolic risk across all sex and gender groups, with a clear dose-response relationship evident after 2 h per day [97]. 

### 3.2. Ectopic Fat

The subsequent failures of subcutaneous and visceral fat to increase lipid storage capacity under the conditions of excessive energy reserves culminates in ectopic partitioning of fat to other organs and tissues, such as muscle and liver. Excess fat can be deposited intracellularly in the form of triglycerides as observed in liver hepatocytes under the condition of the nonalcoholic fatty liver disease (NAFLD) or alternatively deposited into newly developed adipocytes that infiltrate the parenchyma of other organs, such as pancreas [98]. Subsequent ballooning of the cells and infiltration of the organ tissues with inflammatory cells leads to a more aggressive presentation, including steatosis and/or fibrosis. Perilipin 2 (Plin2), a major protein found on the surface of the oil bodies, was shown to be one of the major players in this process [99]. Dysregulated ectopic fat tissue is believed to be the factor that promotes and sustains insulin resistance, thus being a dominant regulator of glucose and lipid metabolism [100]. 

**Figure 1:**
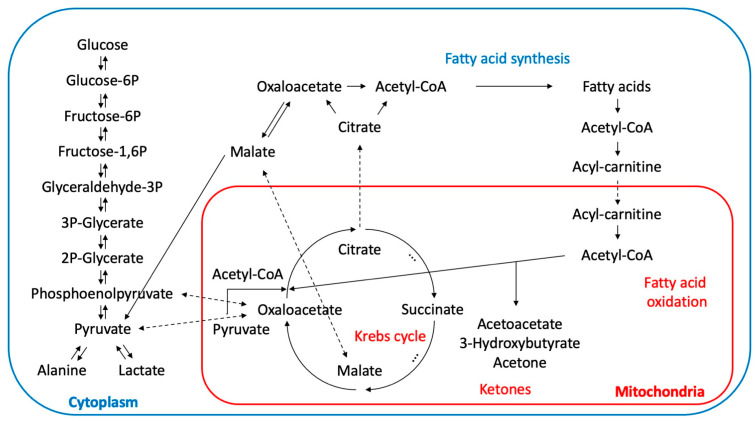
Schematic representation of major pathways of energy metabolism. Pyruvate (rapidly interconverted to lactate and back) forms a primary energy metabolism node with phosphoenolpyruvate and oxaloacetate that connects carbohydrate, lipid, and protein metabolism. Pyruvate, citrate, and malate can be shuffled between cytosol and mitochondria to change the direction of metabolic fluxes.

Local paracrine affects the visceral cavity as expected for mesenteric, pararenal, and epicardial fat depots and could be partially responsible for detrimental metabolic effects associated with the expansion of visceral fat; however, they remain virtually not explored. Direct and potentially continuous delivery of gluconeogenic substrates (Figure 1), such as fatty acids and glycerol [101], from the mesenteric fat into the portal circulation and therefore, to the liver, hold potential to sustain liver tissues in the glucose-producing state, despite the actual fed status of the body. In these metabolic states, individual diversity of obese phenotypes is driven by their ability to sustain higher subcutaneous adipogenesis (a larger number of smaller adipocytes in the adipose tissues), with less visceral adiposity and ectopic lipid accumulation [102]. Overfeeding young subjects by 40% of their energy requirement for only 8 weeks caused a 7.6 kg weight gain, induced visceral and hepatic lipid deposition and mild hepatic and skeletal muscle insulin resistance, and triggered systemic and skeletal muscle inflammation [100].

The reduced capacity to oxidize fatty acids in the muscle due to disturbances in mitochondrial metabolism (metabolic inflexibility) [103] is one of the reasons of ectopic fat accumulation in skeletal muscle tissues. In healthy states, the insulin-driven uptake of lipids triggers their acylation with CoA and transfers into mitochondria for beta-oxidation (Figure 1). In insulin-resistant states, it triggers the inhibition of lipolysis, the accumulation of diacylglycerides, and the slower esterification of free fatty acids [104]. While skeletal muscle gene expression profiles remained similar for triacylglyceride synthesis (DGAT1/2, GPAT1) and ceramide synthesis (SPTLC1/2, ASAH2, CERK), oxidative metabolism was clearly reduced (mCPT1, PGC1α, PPARα/δ, SDHB, NDFU5B) [104]. Acetyl-CoA, derived from beta-oxidation or pyruvate oxidation (Figure 1), is the only fuel that enters the Krebs cycle and cannot be transported outside of mitochondria. For this reason, the Krebs cycle can be broken at certain points as citrate can be transferred to cytosol to increase fatty acid and cholesterol synthesis, while malate can be transferred to cytosol and promote gluconeogenesis via conversion to oxaloacetate. 

Somewhat surprising, lactate derived from anaerobic glycolysis showed the major contribution to metabolic fluxes through the Krebs cycle in all tissues except the brain [105] (Table 2). Low metabolic flaxes of citrate, succinate, and malate may suggest that circulating levels of these metabolites represent the long-term trends of energy metabolism and the net energy balance in the body. The quantitative relevance of these circulating metabolic intermediates as fuels remains unclear and warrants further investigation. 

## 4. Contribution of Gastrointestinal Microbiota and Inflammation to Obesity

Dietary factors, the intestinal microbiota that partially digests them [106], and their metabolites [107] shape visceral fat accumulation (mesenteric fat), intestinal permeability and activation, the transient migration of immune cells [108], and intestinal hormone responses. Understanding these pathways may provide additional clues towards high diversity and individual variability of obese phenotypes. 

### 4.1. Microbiome in Obesity

The human body hosts a complex community of microorganisms, such as bacteria, fungi, protozoa, and viruses, that makeup the microbiome. The gastrointestinal microbiome is dominated by bacteria, the majority of which inhabit the colon at a concentration range of 10^9^–10^12^ CFU/mL [109]. The model minimal microbiome includes seven major phyla and one hundred seventy-one microbial genomes: *Firmicutes* (104), *Bacteroidetes* (29), *Proteobacteria* (22), *Actinobacteria* (12), *Fusobacteria* (2), *Verrucomicrobia* (1), and *Euryarchaeota* (1) [110]. Trace phyla may further include *Lentisphaerae*, *Spirochaetes*, *Thermotogae*, *Synergistetes*, *Tenericutes*, *Elusimicrobia*, and *Cyanobacteria* [111]. As gut microbiota affects nutrient acquisition and energy harvest and feeds metabolites into a wide variety of host metabolic pathways, the effective matching of metagenomic and metabolite profiles of the intestinal microbiome is critically required to understand these contributions [112].

**Figure 2:**
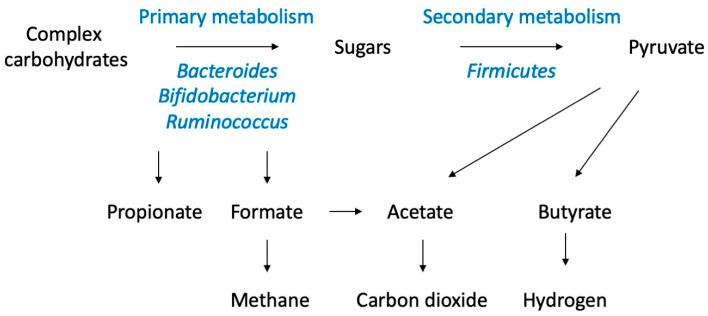
Microbiota-mediated metabolism of complex carbohydrates. Different groups of bacteria preferentially utilize polymeric, oligomeric, monomeric carbohydrates and short-chain fatty acids to complete the fermentation process in the gastrointestinal lumen (adapted from [106]). Variations in microbiome composition, substrate availability, luminal oxygen, and inflammatory status of the gastrointestinal tissues are expected to contribute to the diversity of the metabolic outcomes.

Age, race, geographical location, and ethnicity often present with variations in gut microbiota, which are larger than interpersonal differences and may be attributed to dietary patterns, food composition, and its availability [113]. The major source of energy harvested and used by the gut microbiome are simple and complex carbohydrates that were not digested by the host organs (Figure 2). In addition to the short-chain fatty acids (propionate, acetate, butyrate) that can be absorbed and metabolized by enterocytes, these carbon fluxes can be further fermented into methane, carbon dioxide, and hydrogen [114]. Once again, pyruvate remains as a central node that connects different energy pathways, and the contributions of bacterial lactate and metabolites of Krebs cycle to the host metabolism are poorly understood. 

Bacterial fermentation of complex carbohydrates as a part of plant-based dietary patterns that are especially evident in vegetarian or vegan diets contributes to lower pH levels of the intestinal lumen and feces [115]. Healthy fermentation also rapidly depletes luminal oxygen to promote a proper anaerobic environment in the gut that can be altered through increased inflammation and blood flow in the intestine [116] as well as high-fat diets [117]. Dietary fats also stimulate the production of bile acids that promote the emulsification and absorption of lipids, cholesterol turnover, and the harvest of the fat-soluble vitamins. Bile acids, however, also present with selective antimicrobial properties and typically enrich gut microbiota with bile-acid-tolerant *Bacteroides*, *Alistipes*, and *Bilophila*. Both obese *ob*/*ob* mice as well as obese human subjects had marked reductions in *Bacteroidetes* and compensatory increases in *Firmicutes*, thus showing abnormal *Bacteroidetes*/*Firmicutes* ratios [118]. In the absence of the gut microbiota, however, germ-free mice do not develop obesity despite consuming high-carbohydrate or high-lipid diets, suggesting that the microbiome is critical for excessive energy harvest and retention [119].

Metabolic fates of different short-chain fatty acid metabolites are different, suggesting that certain microbiome profiles and host–microbiome interactions may be more beneficial than others. While bacteria-derived butyrate has a rapid turnover flux similar to 3-hydroxybutyrate and are mostly consumed in situ to support colonic epithelium, propionate is targeted by the liver into the gluconeogenesis pathway, and acetate feeds into lipid metabolism [120]. Likewise, the metabolism of unassimilated amino acids and dietary phenolic acids yields glycine conjugates of phenylpropionic and glutamine conjugates of phenylacetic acids as a part of the xenobiotic detoxification response and detoxification of waste nitrogen [107]. The intestinal epithelial tissues also express a wide variety of the extraoral bitter taste receptors family (T2R, TAS2R) that do not support the bitter sensing in the gut but act as chemosensors to detect many dietary and microbiota-derived metabolites, such as N-acyl-homoserine lactones [121], that modulate host–microbiome interactions, immune responses, and nutrient absorption [122]. 

### 4.2. Inflamamtion in Obesity

Inflammatory cells that infiltrate the hypertrophic adipose tissue find themselves in the ischemic environment that promotes their polarization towards pro-inflammatory states. Both lipid-overloaded adipocytes and associated inflammatory cells provoke insulin resistance [123] in an effort to gain excess to more rapid but less efficient, energy fluxes that are generated by glycolytic pathways and not oxidative phosphorylation (Figure 1). Metabolic dysfunction therefore expresses itself as a low-grade inflammation, insulin resistance, and metabolic homeostasis disruption [124]. These signals can be further modified by the adipose tissue adipokines. Expanding adipose tissue releases different levels of leptin and adiponectin, with leptin levels generally associated with total fat mass, and adiponectin levels loosely associated with increased subcutaneous depots [125].

**Figure 3:**
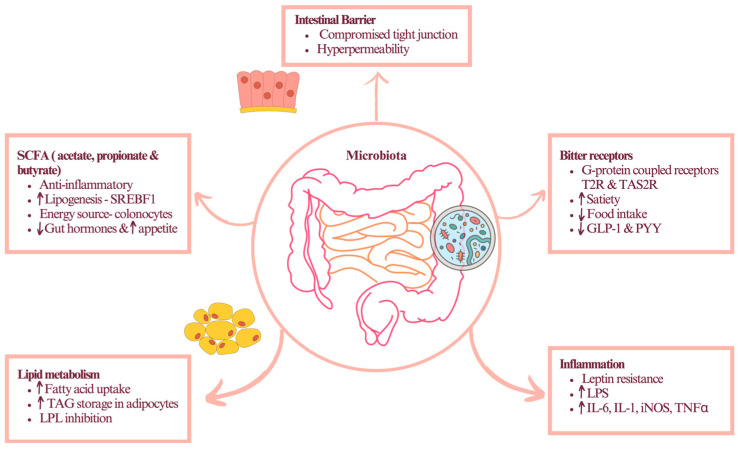
Complexity of contributions of the gastrointestinal system to development of multiple obesity phenotypes. Variations in microbiome-derived short-chain fatty acids, intestinal permeability, dietary metabolites, inflammatory signals, and chemoreceptors responsible for sensing nutrient and immune status of host-microbiome interactions all contribute to complexity of gastrointestinal signaling in obesity. Up (↑) and down (↓) arrows indicate increases or decreases in the levels of the indicated metabolites, respectively.

Inflammation of the adipose tissues promotes de novo lipogenesis, reduces fat oxidation in mitochondria, and accumulates ceramides and diacylglycerides in the metabolically active cells that inhibit insulin signaling [126]. Similar to metabolic mediators, inflammatory cytokines, such as TNF-α, IL-6, and IL-1β, also impair the insulin-signaling pathway, leading to insulin-resistant metabolic conditions [127]. These pro-inflammatory signals circulate to the periphery, where they affect the liver to increase infiltration with resident Kupffer cells and skeletal muscle to promote M1 macrophage infiltration [128]. The gastrointestinal tract that represents a major component of the immune system responds similarly by increasing intestinal perfusion, reducing the integrity of the epithelial barrier, and enhancing recognition of the food and microbial antigens that diffuse from the intestinal lumen [129]. Together, this leads to a highly variable gastrointestinal inflammatory response that depends on the part of the gastrointestinal tract involved (Figure 3). As mesenteric fat is closely associated with the gastrointestinal tissues and feeds its metabolites directly into the portal circulation, the process is likely to sustain liver tissues in the glucose-producing state, thus contributing to hyperglycemia and oxidative stress. Activated immune cells achieve faster energy flaxes by lowering their metabolic efficiency and relying nearly exclusively on the faster glycolytic reactions in the cytosol [130]. 

## 5. Conclusions

The etiology of obesity is very complex, with individual genetic, metabolic, microbiome, and lifestyle factors defining multiple pathophysiological states of this disorder. To achieve and sustain meaningful weight loss as well as support individuals with different obesity types, we need to understand the molecular mechanism behind this classification. Variations in fatty acids oxidation, metabolic fluxes of different energy substrates with a particular focus on pyruvate-phosphoenolpyruvate-oxaloacetate node, high turnover rates of lactate, and extracellular accumulation of Krebs cycle metabolites, such as citrate, emerged as the most promising metabolic targets that allow personalization of obesity and warrant further investigations. 

The additional modulation of gut microbiota [119] and brain axis [131] through dietary interventions, pharmacological treatments, and surgery provides a unique set of tools to individually address multiple obesity phenotypes. For example, phenotype-guided pharmacotherapy with phentermine-topiramate (hungry brain), bupropion-naltrexone (emotional hunger), liraglutide (hungry gut), and low-dose phentermine plus resistance training (slow burn) shows a very interesting recent example of such an approach [132]. Given that the existing body weight is actively maintained and protected, we need to understand how to overcome the multiplicity of the obesity mechanisms and metabolic fluxes that sustain them. 

## Figures and Tables

**Table 1:** Energy reserves available to a 70 kg male (kcal, after [77]).

Tissues	Carbohydrates (Glucose, Glycogen)	Proteins (Mobilizable)	Fats (Triacylglycerols)
Liver	400	400	450
Muscle	1200	24,000	450
Adipose	80	40	135,000
Blood	60	0	45
Brain	8	0	0

**Table 2:** Turnover fluxes for selected circulating carbon metabolites (adapted from [105]).

Metabolite	Flux Rate (nmol g^−1^ min^−1^)
Lactate	374.4 ± 112.4
Glucose	150.9 ± 46.7
Acetate	72.7 ± 17.5
Alanine	70.2 ± 5.4
Pyruvate	57.3 ± 14.2
Glycerol	53.3 ± 2.1
Glutamine	45.6 ± 4.7
3-Hydroxybutyrate	43.3 ± 17.1
Palmitic acid	24.6 ± 4.2
Citrate	16.2 ± 6.6
Succinate	3.1 ± 1.1
Malate	2.0 ± 0.4
